# Bloodmeal sources and feeding behavior of anopheline mosquitoes in Bure district, northwestern Ethiopia

**DOI:** 10.1186/s13071-021-04669-7

**Published:** 2021-03-19

**Authors:** Tilahun Adugna, Delensaw Yewhelew, Emana Getu

**Affiliations:** 1 Debre Tabor University, P.O. Box 272, Debre Tabor, Ethiopia; 2 Jima University, P.O. Box 378, Jima, Ethiopia; 3 Addis Ababa University, P.O. Box 2003, Addis Ababa, Ethiopia

**Keywords:** *Anopheles arabiensis*, *An. coustani*, Human blood index, Host preference index

## Abstract

**Background:**

Mosquito bloodmeal sources determine the feeding rates, adult survival, fecundity, hatching rates, and developmental times. Only the female *Anopheles* mosquito takes bloodmeals from humans, birds, mammals, and other vertebrates for egg development. Studies of the host preference patterns in blood-feeding anopheline mosquitoes are crucial to determine malaria vectors. However, the human blood index, foraging ratio, and host preference index of anopheline mosquitoes are not known so far in Bure district, Ethiopia.

**Methods:**

The origins of bloodmeals from all freshly fed and a few half-gravid exophagic and endophagic females collected using Centers for Disease Control and Prevention light traps were identified as human and bovine using enzyme-linked immunosorbent assay. The human blood index, forage ratio, and host feeding index were calculated.

**Results:**

A total of 617 specimens belonging to *An. arabiensis* (*n* = 209), *An. funestus* (*n* = 217), *An. coustani* (*n* = 123), *An. squamosus* (*n* = 54), and *An. cinereus* (*n* = 14) were only analyzed using blood ELISA. Five hundred seventy-five of the specimens were positive for blood antigens of the host bloods. All anopheline mosquitoes assayed for a bloodmeal source had mixed- rather than single-source bloodmeals. The FR for humans was slightly > 1.0 compared to bovines for all *Anopheles* species. HFI for each pair of vertebrate hosts revealed that humans were the slightly preferred bloodmeal source compared to bovines for all species (except *An. squamosus*), but there was no marked host selection.

**Conclusions:**

All anopheline mosquitoes assayed for bloodmeal ELISA had mixed feeds, which tends to diminish the density of gametocytes in the mosquito stomach, thereby reducing the chance of fertilization of the female gamete and reducing the chances of a malaria vector becoming infected. Moreover, *An. coustani* was the only species that had only human bloodmeals, meaning that this species has the potential to transmit the disease. Therefore, combination zooprophylaxis should be reinforced as a means of vector control because the study sites are mixed dwellings.

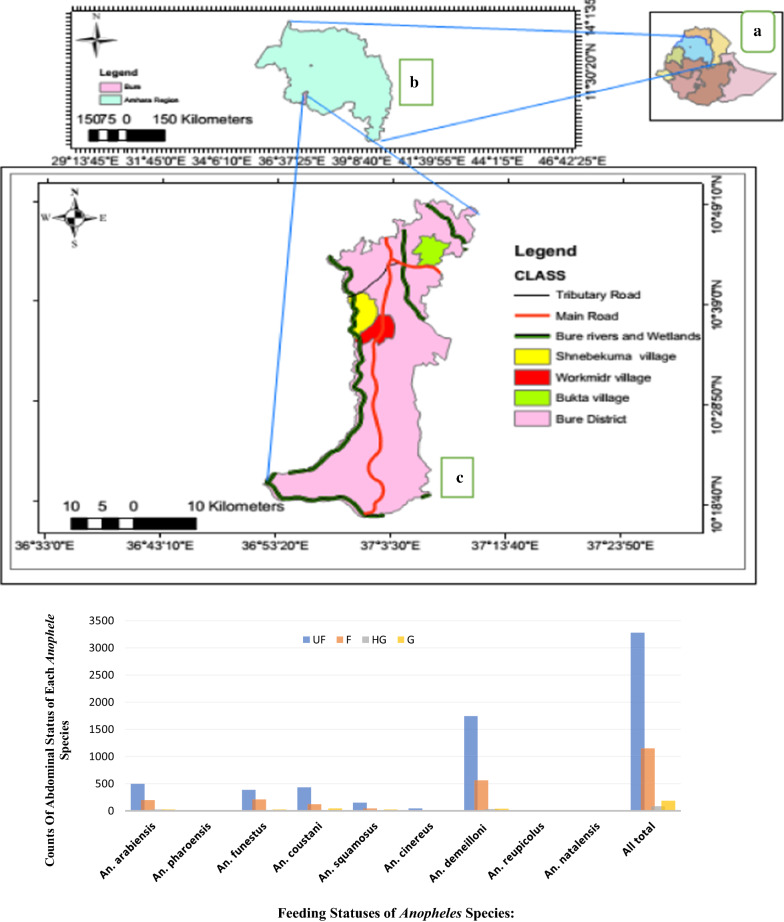

## Introduction

Malaria is transmitted by the blood feeding of infectious female *Anopheles* mosquitoes [1, 2] and has a complex parasite life-cycle, which depends on both humans and mosquitoes [3, 4]. In Ethiopia, malaria is the leading health problem [5] because three-fourths (75%) of the total area of the country is malarious and more than two-thirds (approximately 68%) of the total population lives below 2000 m altitude [5]. Amhara is one of the many regions in the country, and malaria remains a major public health problem there. Bure is one of the approximately 15 districts in the region and carries > 13% of the malaria burden [6]. Across the country, the nature of malaria transmission is seasonal and unstable [7], varying with elevation, temperature, and rainfall [8, 9].

In Ethiopia, over 42 species of *Anopheles* have been identified [10, 11], but *Anopheles arabiensis* is the principal malaria vector while *An. pharoensis*, *An. funestus*, and *An. nili* are secondary vectors [12, 13]*.* Therefore, understanding of the biology and behavior of *Anopheles* mosquitoes can help to understand how malaria is transmitted and can aid in designing appropriate control strategies [14]. Each species of *Anopheles* has its own blood-feeding pattern, host preference, biting, flight range, and host selection behavior [15, 16].

The blood-feeding behavior of malaria vectors is an important parameter in malaria epidemiology [3]. This behavior can influence vectorial potential [1], depending on the vertebrate host groups with which the mosquito makes contact, and influence the spatial distribution of a disease [17]. The most successful malaria vectors commonly feed on humans and secondarily on cattle and other domestic animals, depending on host availability [3]. Host choices and subsequent feeding success depend on host availability [15, 18] including host accessibility, density, host defense mechanisms, host size, proximity to mosquito habitats [19, 20], environmental factors, flight behavior, and feeding periodicity of the mosquitoes [21]. Interventions through long-lasting insecticide-treated nets (LLINs) and insecticide residual spraying (IRS) determine the successful feeding and oviposition nature of malaria mosquitoes [22].

Preference of anophelines to feed on humans can be estimated using the human blood index (HBI). HBI represents the proportion of bloodmeals derived from humans by mosquito vectors [23]. Study of the host-feeding pattern is an essential part of understanding the epidemiology of diseases transmitted by arthropods [24, 25]. Host preference studies have also been used to monitor the effectiveness of vector control programs by observing a reduction in blood-feeding behavior and have served as evidence of control failure [26–28].

Anophelines exhibit a wide range of host preferences, including humans, cattle, sheep, horses, pigs, dogs, cats, other mammals, birds, and reptiles [29–31]. Particularly animal-feeding vectors are known to suppress human bloodmeal sources and reduce the level of infection in the local vector population [32, 33]. However, HBI results do not always reflect host preference [34, 35]. Therefore, several authors have proposed different indices to separate preferential versus opportunistic feeding patterns of mosquitoes [24, 36]. The forage ratio (FR) measures host selection patterns, i.e. quantifies vector selection of a particular vertebrate host rather than other available hosts [34]. It only shows the attributes of one host preference [23] and does not require a full host census [37]. The other parameter is the feeding index (FI), which compares the observed proportion of blood feeds from one host to another host divided by the expected comparative proportion of feeds on the two hosts [17, 24].

Generally, the knowledge of the HBI, blood-feeding preferences, and pattern of a mosquito species provides insight into its vector potential [17, 38] and the epidemiology of disease transmission [24, 25, 29, 39] and allows designing and implementing efficient strategies for vector control [23, 29, 30]. For our study, the HBI, FR, and host preference index (HFI) of anopheline mosquitoes have not been known so far, so we aimed to determine the abdominal status, HBI, FR, and HFI of anopheline mosquitoes in Bure district, northwest Ethiopia.

## Materials and methods

### Study area

**Fig. 1:**
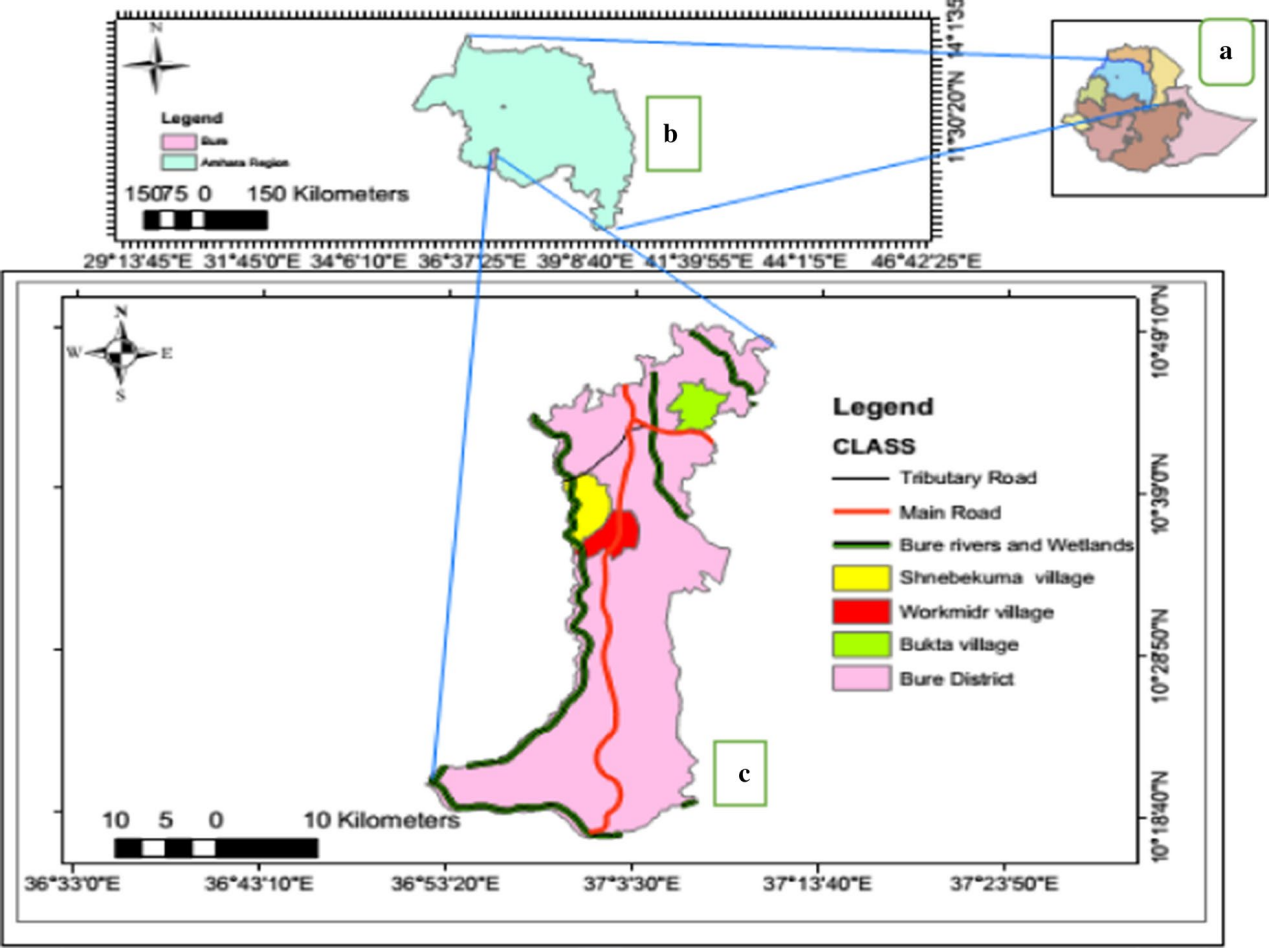
Map of the study area. **a** Ethiopia, **b** Amhara region, and **c** Bure district

A longitudinal study was conducted in Bure district, northwestern Ethiopia, from July 2015 to June 2016. Geographically, Bure district is situated at an altitude ranging from 700 (Blue Nile gorge) to 2350 m above sea level (Fig. 1). Socioeconomically, the majority (85%) of the population is farmers who grow maize, teff (*Eragrostis teff*), pepper, potatoes, wheat, and millet, followed by beans and peas, sunflowers, niger, spices, vegetables, and others. The rest of the population includes merchants (6.8%) and others (non-governmental organizations, civil servants) (8.2%). Animals such as cattle, sheep, hens, mules, and donkeys are reared by most farmers. The proportions of the animals reared in the study district are described in Table 2. In addition, there are both modern and traditional beekeepers. Most of the population in the district lives in houses made of mud with corrugated iron roofs. The mud houses are partly smooth and partly rough. Some of them are painted. The doors and windows of the houses do not have mosquito screening. In each farmer’s compound, there is a separate kitchen and latrine house. The distance between farmers' houses is 10–15 m. Both humans and animals live inside the same house.

Most of Bure district has a subtropical zone (Woina-Dega) climate with annual mean minimum and maximum temperature of 9.9 °C and 29.2 °C, respectively, and 2000 mm mean annual rainfall ranging being 1350–2500 mm. The major rainy season of the district is from July to September, and a small amount falls from May to June and from October to December. The other months (January–April) are dry seasons [40].

The study was conducted in three rural villages, Bukta, Workmidr, and Shnebekuma, from July 2015–June 2016. The detailed description of the three villages is provided elsewhere [41]. These villages are malarious. Bed nets have been distributed to the three villages once every 3 years before malaria infestation begins, in the first week of September. Moreover, anti-malaria chemical spraying (IRS) (Deltamethrin, K-Othrine Flow) has been administered in the three villages according to the national spraying operation guidelines [12].

### Adult mosquito collection, identification, and processing

#### Mosquito collection

*Anopheles* mosquitoes were collected each month from July 2015–June 2016 using Centers for Disease Control and Prevention light trap catches (LTCs), pyrethrum spray catches (PSCs), and artificial pit shelters (APSs). In each village, nine houses for LTCs and ten houses for PSCs were randomly selected, distributed near the breeding sites, in the middle and on the periphery of the village. Similarly, nine LTCs were prepared to collect outdoor host-seeking mosquitoes in each village. In addition, six APSs were prepared in three villages to collect outdoor resting mosquitoes; each village had two.

Indoor host-seeking *Anopheles* mosquitoes were collected from 6:00 p.m. (sunset) to 6:00 a.m. (sunrise) using LTCs (Model 512; J. W. Hock Co., Atlanta, GA, USA) once per month per house for 1 year [42, 43]. Likewise, the outdoor host-seeking mosquitoes were collected by LTCs from 06:00 p.m. to 06:00 a.m. once per month. Indoor-resting mosquitoes were collected in the mornings from 6:00 a.m. to 8:30 a.m. using PSCs for 1 consecutive year. Collection was carried out using white floor sheets, hand lenses, baygon aerosol (Tetramethrin: 0.4% and Permethrin: 0.4%; SC. Johnson & Sons Inc., USA), small Petri dishes, paper cups with net covers, forceps, cotton wool, and a torch [44].

Additionally, outdoor-resting mosquitoes were collected in the morning from 6:30 a.m. to 7:30 a.m. using APSs by handheld mouth aspirator. APSs were constructed under the shade of various dense shrub trees 10–15 m away from the residential villages. Before collection began, the mouth of each pit shelter was covered with insecticide-untreated white net to prevent mosquitoes from escaping and for visibility purposes. Resting mosquitoes were collected for about 10–20 min in each pit [45]. The number of human occupants and other potential vertebrate hosts in each surveyed house during the previous night was recorded (Table 2). Moreover, the condition of each surveyed house was recorded, including the type of house, type of walls, number of long-lasting insecticide-treated nets (LLINs) used, and spray status.

Collection of mosquitoes was carried out after obtaining ethical approval from the ethics review committee of Addis Ababa University (reference no. CNSDO/382/07/15), Amhara Health Regional Bureau (permission reference no. H/M/TS/1/350/07), and the Head of the Bure District Health Office (permission reference no. BH/3/519L/2). Moreover, informed consent was obtained from the heads of the selected households.

#### *Anopheles* mosquito species identification

Mosquitoes collected by LTCs, PSCs, and APSs were identified morphologically at the genus level using taxonomic keys [46, 47]. In addition, morphologically identified and separately stored *An. gambiae* specimens were identified by species-specific PCR [48] at the Molecular Biology Laboratory of Tropical and Infectious Diseases Research Center, Jima University. Then, DNA was extracted from individual preserved *An. gambiae* complex species based on DNeasy Blood and Tissue Kits [49]. DNA amplification was then carried out followed by gel electrophoresis [48]. Finally, agarose gel was placed on the UVP. Those mosquitoes that remained unamplified were tested three times independently.

### Survey of vertebrate hosts

Human and domestic animal (hens and mammals) census reports were obtained by interviewing the heads of households during house-to-house visits in the district. The number of humans and domestic vertebrates in neighboring houses was not counted. Potential blood hosts for *Anopheles* mosquitoes from PSCs were not included because of the presence of fewer engorged *Anopheles.*

### Determination of abdominal status of *Anopheles* mosquitoes

The abdominal conditions of *Anopheles* were determined based on blood digestion and ovarian development using standard keys as unfed, freshly fed, half-gravid, and gravid [50]. Finally, freshly fed and half-gravid anophelines were taken to Jima University for bloodmeal ELISA (enzyme-linked immunosorbent assay) tests.

### Identification of the bloodmeal sources of *Anopheles* mosquitoes

The origins of bloodmeals of all freshly fed and a few half-gravid female *Anopheles* mosquitoes (*An*. *arabiensis*, *An. funestus*, *An. coustani*, *An. squamosus*, and *An. cinereus*) collected using LTCs were identified as human and bovine using ELISA [51]. Each mosquito abdomen was ground with 100 μl of phosphate-buffered saline (PBS) using an electrical pestle. The pestle was rinsed with 100 μl PBS to make a total of 200 μl final volume, and 100 μl homogenate was added to 96-well ELISA plates. Similarly, 100 μl animal sera (1/100 in PBS) and 100 μl unfed female adult *Anopheles* mosquitoes of each species (from a laboratory colony) were added to 96-well ELISA plates as a positive and negative control, respectively. Moreover, 100 μl PBS was used alone as a negative control. Then, the plates were covered and incubated at room temperature for 2 h. After incubation, the well contents were discarded, and they were tapped upside-down five times on tissue paper and washed three times with 200 μl PBS-Tween-20 using ELISA washer. Then, 50 μl human peroxidase conjugate (lot no. 023M4782; batch no. 023M4782; product no. A0170) was added; plates were covered and incubated for 1 h at room temperature. Plates were washed with ELISA washer three times with 200 μl PBS-Tween-20, and 100 μl of ABTS was added to each well and incubated for 30 min for human blood detection.

To bovine blood sources, 50 μl bovine phosphatase conjugate (lot no. 062M4761V/Sigma-Aldrich.com) was added and then covered and incubated for 1 h at room temperature. The wells were washed three times with 200 μl PBS-Tween-20 with ELISA washer, and 100 μl pNPP (catalog no. 0421-01; lot no. H4014-VG96) substrate was added to each plate and incubated for 1 h. Finally, positive samples, including positive control, were changed to blue-green color for human blood (peroxidase) and dark yellow reactions (phosphatase) for bovine blood (detected visually). Immediately, using the ELISA reader, the value of each plate was determined at 405 nm wavelength. Samples were considered positive if absorbance values exceeded two times the mean of three negative controls, unfed mosquitoes/PBS-blank solution.

### Data analysis

Data were entered and cleaned using Microsoft Excel 2007 and analyzed using the SPSS software package, version 20.0 (SPSS, Chicago, IL, USA). Before applying mean comparison, normality of bloodmeal sources (host types), HBI, and BBI data were checked, and data were log transformed [log 10 (*x* + 1)]. The HBI and bovine blood index (BBI) were calculated as the proportion of mosquitoes that fed on human and bovine bloodmeals out of the total bloodmeals determined/tested [23]. Mixed (human + bovine) bloodmeals were added to the number of a human and bovine bloodmeals when calculating the overall HBI and BBI. The presence of significant differences between HBI and BBI and indoor and outdoor HBI/BBI was checked by independent *t*-test (*p* < 0.05). Variation among bloodmeal sources (host types) for *Anopheles* mosquitoes was separated by one-way ANOVA. The Tukey HSD test was run for mean separation variation (in ANOVA) (HSD) (*p* < 0.05). Statistical test significance was considered at *p* < 0.05 during the analysis.

Foraging ratios (FRs) were determined to obtain the proportion of bloodmeals taken only from humans and cattle. FRs were calculated as the percent of female *Anopheles* mosquitoes (five species as described in the Results section) containing blood of a particular host divided by the percent of the total available host population represented by the particular host [36] as follows:

$${\text{FR}} = \left( {{\text{NAE}}/{\text{NTE}}} \right)/({{\text{NAP}}}/{{\text{NTP}}})$$

where FR = the foraging ratio of *Anopheles* species, NAE = number of engorged female mosquitoes containing blood from host-1, NTE = total number of engorged females, NAP = number of type 1 hosts in the collection area, and NTP = total number of hosts of all types in the collection area.

A foraging ratio of 1 indicated neither a selective bias nor avoidance of a particular host animal (opportunistic = equally feeding); FRs significantly > 1 indicated a selective bias, and values < 1 indicated avoidance of a host in favor of other available hosts [24, 36]. However, in our study, the percentage of FRs was only calculated for humans and cattle, and comparison was made between the two hosts. The host preference indices (HFI) is defined as the observed proportion of feeding on one host compared to another divided by the expected comparative proportion of feeds on these two hosts [17, 24]; the formula is as follows:

$${\text{HFI}} = \left( {Nx/Ny} \right)/\left( {Ax/Ay} \right)$$

where ‘*Nx’* and ‘*Ny’* are the mean numbers of bloodmeals taken from hosts ‘*x*’ and ‘*y*’ per study site, respectively, and ‘*Ax’* and ‘*Ay’* are the mean numbers of hosts ‘*x*’ and ‘*y*’ per study site, respectively. An index of 1 indicated equal feeding on the two hosts. HFI > 1 indicated that host ‘*x*’ was preferentially fed upon, whereas a value < 1 indicated that host ‘*y*’ was preferentially fed upon [17]. HFIs were calculated for each pair of hosts (humans: cattle) [24].

## Results

### Abdominal status of female *Anopheles* mosquitoes

**Fig. 2:**
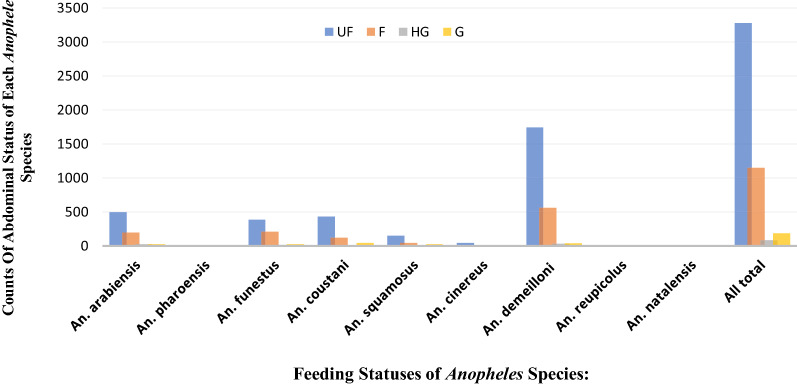
Abdominal statuses of *Anopheles* mosquito species in Bure district, Ethiopia. UF, unfed; F, fed; HG, half gravid; G, gravid

The overall abdominal status of each adult female *Anopheles* mosquito is presented in Table 1 and Fig. 2. Of 4703 *Anopheles* mosquitoes collected, a higher proportion of mosquitoes were unfed (69.7%), followed by fed (24.5%), gravid (3.9%), and half-gravid (1.9%).

**Table 1:** Abdominal status of female *Anopheles* mosquitoes by place and method of collection in Bure district, Ethiopia, from July 2015 to June 2016

Blood digestion stage	Place of collection			% Abdominal status by method of collection			
	Indoor	Outdoor	Total (%)				
	(LTs and PSCs), *n* (%)	(LTs), *n (*%)		LTs, *n* (%)	PSCs, *n* (%)	APTs, *n* (%)	Total (%)
UF	1023 (31.2)	2253 (68.8)	3276 (69.7)	3267 (99.7)	9 (0.3)	0	3276 (69.7)
F	646 (56.0)	507 (44.0)	1153 (24.5)	1145 (99.3)	8 (0.7)	0	1153 (24.5)
HG	39 (43.8)	50 (56.2)	89 (1.9)	89 (100.0)	0	0	89 (1.9)
G	89 (48.1)	96 (51.9)	185 (3.9)	183 (98.9)	2 (0.1)	0	185 (3.9)
Total	1797 (38.2)	2906 (61.8)	4703 (100)	4684 (99.6)	19 (0.4)	0	4703 (100)

Unfed (UF), fed (F), half-gravid (HG), and gravid (G)

Overall, 56.0% (*n* = 646/ 1153) fed Anopheline mosquitoes were from indoor and 44.0% (*n* = 507/1153) from outdoor collections. The proportion of half-gravid (HG) mosquitoes collected outdoors (*n* = 50, 56.2%) was greater than that of HG mosquitoes collected indoors (*n* = 39, 43.8%). Collection method comparison indicated that > 99.6% of unfed (UF), fed (F), HG, and gravid (G) were collected by LTCs while the remaining catches were by PSCs. However, APSs was not fully productive (Table 1). Because of the unsuccessful catches by PSCs and APS, the degrees of exophily and endophily behavior were not determined.

### Compositions and abundances of potential vertebrate hosts for *Anopheles* mosquitoes

A total of 3803 hosts were recorded from 324 surveyed houses in three villages. Of these, hosts from Bukta accounted for 39.2%, Shnebekuma 33.0%, and Workmidr 27.8%. Hosts included: bovines (40.0%), humans (37.7%), sheep (16.0%), donkeys (0.8%), mules (0.7%), chickens (4.0%), and dogs (0.7%). Of these, a higher proportion of human and cattle hosts was recorded in the study area (F_6, 77_ = 160.863; *p* = 0.001) (Table 2).

**Table 2:** Mean difference between hosts

Identified hosts	(M ± SE)
Humans	4.419 ± 0.07^a^
Bovines (cattle)	4.681 ± 0.100^a^
Sheep	1.881 ± 0.093^b^
Donkeys	0.166 ± 0.045^b^
Mules	0.180 ± 0.046^b^
Hens	1.417 ± 0.252^b^
Dogs	0.2658 ± 0.05973^b^

### Distribution of bloodmeals across villages

As indicated in Table 3, 609 (collected by LTs) *Anopheles* mosquitoes were assayed for bloodmeal source analysis using ELISA. Most of them had fed (*n* = 575, 94.4%); however, the distribution was not equal, and a large proportion was from Shnebekuma (76.5%). In the study area, both humans and animals were living in the same houses. Of these, most mosquitoes had a mixed bloodmeal source (human and bovine) (88.8%, *n* = 541). The proportion of mixed blood meals was greater than that of single bloodmeals (either human or bovine) across the three villages. However, a higher proportion of mixed blood (74.4%, *n* = 419/541) was recorded in Shnebekuma.

**Table 3:** Distribution of bloodmeal sources of *Anopheles* mosquitoes in the three villages using LTs in Bure, Ethiopia, from July 2015 to June 2016

Villages	Total numbers of tested mosquitoes (%)	Total tested positive mosquitoes (%)	Only human blood positive (%)	Only hovine blood positive (%)	Mixed
Bukta	88 (14.4)	79 (13.7)	2 (100)	7 (21.9)	70 (12.9)
Workmidr	58 (9.5)	56 (9.7)	0	4 (12.5)	52 (9.6)
Shnebekuma	463 (76.1)	440 (76.5)	0	21 (65.6)	419 (77.4)
All total	609 (100%)	575 (100)	2 (100)	32 (100)	541 (100)

### Bloodmeal indices of *Anopheles* mosquitoes

Table 4 shows the bloodmeal origins and HBI of *Anopheles* mosquitoes by site and collection method. Overall, 617 (LTs and PSCs) *Anopheles* mosquitoes (fed and HG) belonging to five species (*An. arabiensis*, *An. funestus*, *An. coustani*, *An. squamosus*, and *An. cinereus*) were tested by ELISA. Of these, 94.2% (*n* = 581) were positive for host blood antigen and the remaining 5.8% (*n* = 36) were unidentified. From 581 positive samples (LTs and PSCs), the largest proportion (99.0%, *n* = 575) was from LTCs and the smallest proportion (1.0%, *n* = 6) from PSCs. Of 575 positive samples (LTs), the majority (5p7%) was from indoor collection.

**Table 4:** Bloodmeal sources of *Anopheles* mosquito species by site and method of collection in Bure district, Ethiopia, from July 2015 to June 2016

Species	Total tested, negative and positive for blood ELISA				Numbers of tested (by LTs and PSCs) positive for blood antigen (%)			Single bloodmeals (by LTs)				Mixed bloodmeals (both human and bovine bloodmeals)			Overall (single + mixed) numbers and % from LTs only	
	Total tested specimens from		Total LTs		LTs		PSCs	Human only, *n* (%)		Bovine only, *n* (%)		LTs, *n* (%)		PSC, *n* (%)	HBI, *n* (%)	BBI, *n* (%)
			Testedunknow (%)	Tested positive (%)	In	Out	*n* (%)									
					*n* (%)	*n* (%)		In	Out	In	Out	In	Out			
	LTs	PSCs														
*An. arabiensis*	208	1	11 (5.3)	197 (94.7)	124 (59.6)	73 (35)	0 (0.0)	0	0	6 (2.9)	0	118 (56.7)	73 (35.1)	0 (0.0)	191 (91.8)	197 (94.7)
*An. funestus*	213	4	9 (4.2)	204 (95.8)	117 (54.9)	87 (40.8)	4 (100)	0	0	9 (4.2)	0	108 (50.7)	87 (40.8)	4 (100)	195 (91.5)	204 (95.8)
*An. coustani*	122	1	9 (7.4)	113 (92.6)	60 (49.2)	53 (43.4)	1 (100)	2 (1.6)	0	1 (0.8)	4 (3.3)	57 (46.7)	49 (40.2)	1 (100)	108 (88.5)	111 (91)
*An. squamosus*	54	0	3 (5.6)	51 (94.4)	24 (44.4)	27 (50)	0 (0.0)	0	0	0	12 (22.2)	24 (44.4)	15 (27.8)	0 (0.0)	39 (72.2)	51 (94.4)
*An. cinereus*	12	2	2 (16.7)	10 (83.3)	6 (50)	4 (33.3)	1 (50)	0	0	0	0	6 (50)	4 (33.3)	1 (50)	10 (83.3)	10 (83.3)
All total	609 (100)	8 (100)	34 (5.6)	575 (94.4)	331 (54.3)	244 (40.1)	6 (75)	2 (0.3)	0	16 (2.6)	16 (2.6)	313 (51.4)	228 (37.4)	6 (75)	543 (89.2)	573 (94.1)

Of 208 tested *An. arabiensis*, only 94.7% were positive. Of these positive bloodmeals, 91.8% were mixed bloodmeal (LTs-in and out) and 3.0% only of bovine bloodmeal origin. No single *An*. *arabiensis* specimen had blood from human only. However, the indoor and outdoor HBI (*t* = 1.587; *df* = 22; *p* = 0.127), BBI (*t* = 1.406; *df* = 22; *p* = 0.173), and overall HBI and BBI of *An*. *arabiensis* did not show any statistically significant difference between them (*t* = − 0.05; *df* = 22; *p* = 0.961).

The result of this study revealed that from 213 tested *An. funestus* specimens, only 95.8% were positive for bloodmeal ELISA. Of the total positive specimens, 91.5% had mixed bloodmeals (LTs, inside and outside), and the remaining 4.2% had only bovine. From mixed bloodmeals, 54.9% were from indoor and 40.8% from outdoor collection. However, a single bovine bloodmeal was found from indoor collection. The overall (single plus mixed) HBI of *An. funestus* was 91.5%, which was slightly less than that of the overall BBI (95.8%). However, the indoor and outdoor HBI (*t* = 1.322; *df* = 22; *p* = 0.2), BBI (*t* = 1.355; *df* = 22; *p* = 0.189) and overall HBI and BBI of *An. funestus* did not show any statistically significant difference between them (*t* = − 0.168; *df* = 22; *p* = 868).

A total of 122 specimens of *An. coustani* were tested for blood ELISA. Most (92.6%) were positive for blood feeding, and the rest had bovine (4.1%) and human blood meals only (1.6%). Of these, the overall mixed bloodmeal (LTs-in and out) was 86.9%. For all positive samples, 49.2% were from indoor collection and 43.4% from outdoor collection. *An. coustani* was the only species that had only human bloodmeals. However, the indoor and outdoor HBI (*t* = 0.546; *df* = 22; *p* = 0.591) and BBI (*t* = 0.662; *df* = 22; *p* = 0.515) as well as overall HBI and BBI of *An*. *arabiensis* did not show any statistically significant differences between them (*t* = − 0.043; *df* = 22; *p* = 0.966). Similar to this species, both *An. squamosus* and *An. cinereus* did not show any statistically significant difference between the indoor and outdoor HBI and BBI and overall HBI and BBI (*p* > 0.05).

### Foraging ratio and host feeding/preference index of *Anopheles* mosquitoes

The foraging ratio values and feeding preference index of *Anopheles* mosquitoes are presented in Table 5. Humans and cattle were the most common vertebrate hosts in the study area (Table 2). The FR for humans was slightly > 1.0 for all *Anopheles* species. Similarly, the FR for cattle was slightly > 1.0 for all *Anopheles* species. Calculation of the HFI for each pair of vertebrate hosts revealed that humans were the preferred bloodmeal source for bovines for all species (except *An. squamosus*), but there was no marked host selection (Table 5).

**Table 5:** Foraging ratio (FR) and host preference/feeding index (HFI) of *Anopheles* mosquitoes in Bure, Ethiopia, from July 2015 to June 2016

Hosts	Vertebrate Host, *n* (%)	Mosquito species	HBI	BBI	Total FR		HPI human: bovine
					HBI	BBI	
Human	1435 (48.6)	*An. arabiensis*	91.8	–	1.88	–	1.03
Cattle	1520 (51.4)		–	94.7	–	1.84	
Human	1435 (48.6)	*An. funestus*	91.5	–	1.88	–	1.01
Cattle	1520 (51.4)		–	95.8	–	1.86	
Human	1435 (48.6)	*An. coustani*	88.5	–	1.82	–	1.03
Cattle	1520 (51.4)		–	91	–	1.77	
Human	1435 (48.6)	*An. squamosus*	72.2		1.49	–	0.81
Cattle	1520 (51.4)		–	94.4	–	1.84	
Human	1435 (48.6)	*An. cinereus*	83.3	–	1.71	–	1.06
Cattle	1520 (51.4)		–	83.3	–	1.62	

## Discussion

In this study, > 95% of Anopheline mosquitoes were collected by LTCs. This is identical to other findings in which more malaria vectors were trapped while host seeking than resting [52–56]. Of the 4703 collected mosquitoes, most (69.7%) were unfed. Consistent with our study, Fornadel et al. [57] in Zambia, Bashar et al. [58] in Bangladesh, and Getachew et al. [56] in Ethiopia collected mostly unfed *Anopheles* mosquitoes using LTCs. These unfed mosquitoes are stimulated and attracted by the light generated by incandescent bulbs from light traps [58, 59]. As a result, mosquitoes were caught while searching for their bloodmeals before they took blood. However, contrary to this study, Animut et al. [60] collected more freshly fed than unfed *Anophelines* species using light traps in Ethiopia. The catches of more freshly fed mosquitoes using light traps could be due to the recapturing of mosquitoes after repeated feeding behavior [61].

This study revealed that the majority of the bloodmeal sources of *Anopheles* mosquitoes were mixed bloodmeal (both human and bovine) (88.8%, *n* = 541), which was extremely large compared with single bloodmeal sources. Compared with villages, most blood-feeding mosquitoes were from Shnebekuma village (77.4%, *n* = 419). This implies that this village needs more attention than the rest.

The present study showed that no single *An. arabiensis* specimen had a human bloodmeal origin. This is inconsistent with the reports of Massebo et al*.* [62] (8.0%), Animut et al. [60] (33.7%), Yewhalaw et al. [63] (6%), Getachew et al. [56] (50.7%), and Ngom et al. [64] (40.1%) in Ethiopia and Senegal. On the other hand, the presence of single bovine blood origins of *An. arabiensis* (2.9%) was minimal compared to the reports of Massebo et al. [64], Animut et al. [60], Yewhalaw et al. [63], and Getachew et al. [56], which found 7.1%, 38.2%, 23.5%, and 20.9% bovine bloodmeals in Ethiopia, respectively (using LTs). This is probably due to the presence of high human populations as well as cattle for bloodmeal sources, diverting more *An. arabiensis* to feed on both humans and cattle.

In the present study, the majority of *An. arabiensis* had mixed bloodmeals, which was higher compared to other studies [56, 60, 63, 64] that reported 65%, 13.2%, 1.6%, and 4.4% in various parts of south and southwest Ethiopia, respectively. The highest proportion of mixed feeding implies that the sites are mixed dwellings (humans and cattle) [29, 65]. The practice of having humans, cattle, hens, donkeys, mules, etc., in the same house (one house) was confirmed during this survey (personal observation), which contributes to having higher rates of mixed feeding because of the alternative hosts. Moreover, this is also probably associated with the very high incidence of disturbances [34] or climatic factors [58, 65]. Generally, in this study the proportion of mixed bloodmeals was higher than that of single feeding, implying that *An. arabiensis* has plasticity in feeding behavior in the area. Other studies also strengthen this finding [66–69].

The overall HBI and BBI and indoor and outdoor HBI and BBI of *An*. *arabiensis* did not show any statistically significant differences between them, which indicates opportunistic feeding behavior in the area. Similar feeding preferences are reported from southern Ethiopia where people and livestock either share the same houses or where cattle are kept separate but close to houses during the night [60, 70].

This study demonstrated that most *An. funestus* had mixed bloodmeals (humans and cattle) but no single human bloodmeal was detected, similar to *An*. *arabiensis*. The absence of a single human bloodmeal source is in agreement with the findings Massebo et al. [64] but contradicts other studies [71, 72] that reported an extremely high single HBI for *An. funestus* in Kenya (90.8%, 99.5%) and Cameroon (98%), respectively. Therefore, our result indicated that *An. funestus* changes its bloodmeal sources from only humans [67, 73] to both cattle and humans. Various reports have indicated that *An. funestus* now feeds on the blood of humans, goats, calves, chickens, cows, dogs, goats, and equines [66–69], depending on the availability of host types.

The overall (single plus mixed) HBI and BBI and indoor and outdoor HBI and BBI of *An. funestus* did not show any statistically significant differences among them, indicating its opportunistic feeding behavior in the area. However, the overall (single plus mixed) HBI (91.5%) and BBI (95.8%) of *An. funestus* were higher than other findings from Kenya (HBI = 25.2%; BBI = 57.7%) [68] and Ethiopia (HBI = 86.0%; BBI = 14.3%) [74]. These equal proportions of bloodmeal sources in our study were due to the practice of mixed dwelling activities in the three villages. In Ethiopia, *An*. *arabiensis* and *An. funestus* are well-known malaria vectors [12, 13]. In Bure district, these species were found together with *Plasmodium* species [76]. Therefore, the role of mixed dwellings and combination zooprophylaxis should be appreciated to discourage the roles of these species in malaria transmission.

The blood ELISA result of *An. coustani* indicated that the majority (92.6%) of this species had mixed bloodmeals, and the rest had only bovine (4.1%) and human bloodmeals (1.6%). Though proportionally different, Muriu et al. [68] reported 71.4% and 5.4% of *An. coustani* feeding on humans and bovines blood alone in Kenya, respectively. In southwest Ethiopia, Getachew et al. [56]*.* also reported that *An. coustani* had 3.3% (2/59) human and 92.8% (64/69) bovine bloodmeals alone. In our study, *An. coustani* was found with human bloodmeals, which is corroborated by Getachew et al. [56]. On the contrary, Yewhalaw et al. [63] did not detect any *An. coustani* with human blood in southwest Ethiopia. Although this species still was not confirmed as a malaria vector in Ethiopia, many studies in Ethiopia [63, 75, 76], Cameroon [77], and Kenya [78, 79] have reported *An. coustani* with malaria parasites (*Plasmodium* spp.). These results suggest that *An. coustani* is responsible for malaria transmission in Bure district.

Moreover, the FRs for humans were > 1.0 for all of the anopheline species, except *An. squamosus*. Based on the HFIs for each pair of vertebrate hosts, humans were relatively the preferred blood source for all tested species, except for *An. squamosus*. The limitation of this study is that the other bloodmeal sources of *Anopheles* mosquitoes (blood ELISA), such as sheep, donkeys, mules, hens, and dogs, were not determined although they were recorded in the district. Therefore, HFI could not be calculated for the aforementioned vertebrate hosts.

## Conclusions

This animal census survey indicated that humans, bovines, sheep, donkeys, mules, hens, and dogs were the common vertebrate hosts in the study area; however, the proportions of humans and bovines were significantly high. Therefore, *Anopheles* mosquitoes have many alternative bloodmeal sources. Houses were traditional (made of mud) and served for cooking, sleeping, and tethering of livestock, resulting in higher indoor temperatures. Hence, this microclimate attracts more mosquitoes and provides more access to bloodmeal sources, and a relatively high proportion of indoor feeding mosquitoes were recorded.

All the anopheline mosquitoes assayed for blood ELISA indicated the presence of a high proportion of mixed bloodmeals (humans and cattle), which is very important compared to single human meals because mixed feeding tends to diminish the density of gametocytes in the mosquito stomach, thereby reducing the chance of fertilization of female gametes and reducing the chances of malaria vector infection [34, 58, 64, 68, 80]. Moreover, among assayed anopheline mosquitoes, only *An. coustani* had solely human blood, implying that his species may be linked with malaria transmission. Therefore, proper investigation is required to gain certainty about its role as a malaria vector. Further confirmation is needed on whether the existing intervention activity against *An. coustani* is fully effective or not. Combination zooprophylaxis should be reinforced as a means of vector control because the study sites are mixed dwellings.

## Data Availability

All data (generated and analyzed) presented in this study are available from the corresponding author on reasonable request.

## Abbreviations

MoH: Ethiopian Ministry of Health
PMI: President’s Malaria Initiative
LLINs: Long-lasting insecticide-treated nets
IRS: Insecticide residual spraying
HBI: Human blood index
FR: Forage ratio
FI: Feeding index
HFI: Host preference index
WHO: World Health Organization
ELISA: Enzyme-linked immunosorbent assay
CDC: Centers for Disease Control and Prevention
LTCs: Light trap catches
BBI: Bovine blood index
HG: Half gravid
PSCs: Pyrethrum spray catches
APSs: Artificial pit shelters
UF: Unfed
F: Fed
G: Gravid
